# Identifying barriers to compliance with a universal inpatient protocol for *Staphylococcus aureus* nasal decolonization with povidone-iodine

**DOI:** 10.1017/ice.2022.234

**Published:** 2023-07

**Authors:** Rebecca A. Stern, Bryan D. Harris, Mary DeVault, Thomas R. Talbot

**Affiliations:** 1 Division of Infectious Diseases, Department of Medicine, Vanderbilt University Medical Center, Nashville, Tennessee; 2 Department of Infection Prevention, Vanderbilt University Medical Center, Nashville, Tennessee

## Abstract

Academic hospital nurses were surveyed to assess adherence barriers to a universal povidone-iodine nasal decolonization protocol to prevent *Staphylococcus aureus* infection. Low training rates, inadequate supplies, documentation and tracking challenges, patient refusal, and burnout contributed to suboptimal adherence. Prioritizing education is essential but alone is insufficient for successful protocol adoption.

Anterior nares colonization with *Staphylococcus aureus*, noted in up to 30% of the general population, increases the risk of *S. aureus* infection, including healthcare-associated infections (HAIs).^1–4^ Recognizing the impact of healthcare-associated *S. aureus*, the Centers for Disease Control and Prevention (CDC) recommends strategies to reduce *S. aureus* colonization among hospitalized patients. In 2019, these recommendations were broadened to target patients admitted to intensive care units (ICU) and those outside the ICU with a central venous (CVC) or midline catheter.^5^ These recommendations included intranasal decolonization with either iodophor (povidone-iodine, PI) or mupirocin, in conjunction with chlorhexidine (CHG) bathing. PI is an attractive alternative to mupirocin given the concern for mupirocin resistance, similar efficacy in reducing nasal MRSA colonization, and ease of use due to absence of a provider order requirement for use (as PI is an antiseptic versus mupirocin, an antibiotic).^6,7^

Few studies have evaluated challenges to implementation of an intranasal decolonization protocol from the frontline nursing perspective. In January 2021, Vanderbilt University Medical Center (VUMC) adopted a universal standard operating procedure (SOP) for PI nasal decolonization for all patients recommended by the CDC guidelines. Concurrent daily CHG bathing for these patients had been in place since 2018 and was continued. Rates of SOP adherence were 57%–60% from June to November 2021. To better understand the barriers to implementation, we surveyed frontline nursing personnel for attitudes and experience with the povidone-iodine SOP.

## Methods

In March 2022, nurses working in adult inpatient units at VUMC, a 1,000-bed tertiary-care academic hospital, were surveyed using REDCap^®^ (Vanderbilt University, Nashville, TN). Participation was voluntary and anonymous. The survey link and QR code were distributed via e-mail from the chief nursing officer and flyers posted in hospital units. Practices and attitudes regarding intranasal PI use and perceived adherence barriers were evaluated using a Likert rating scale to indicate level of agreement. Domains included provider education, training, resources, and patient identification, education, and acceptance (Table 1). Optional free-text responses were also collected. Statistical comparisons using the 2-sample test of proportions were conducted using Stata version BE 17 software (StataCorp, College Station, TX).

**Table 1.** Frontline Nursing Survey Results on Barriers to Povidone-Iodine Nasal Decolonization Protocol Adherence

Survey Question	Response, No. (%)				
	Strongly Agree	Often Agree	Neither Agree nor Disagree	Often Disagree	Strongly Disagree
**Patient Identification and Education**					
“I have a good understanding as to why we use intranasal PI on our patients.”	122 (49.2)	70 (28.2)	21 (8.5)	21 (8.5)	14 (5.6)
“I can easily identify which of my patients need to undergo intranasal PI.”	105 (42.3)	67 (27.0)	27 (10.9)	34 (13.7)	15 (6.0)
“I am comfortable talking with patients who have questions about the PI application.”	98 (39.7)	81 (32.8)	20 (8.1)	40 (16.2)	8 (3.2)
“My patients understand why intranasal PI decolonization is being performed.”	52 (21.0)	70 (28.2)	39 (15.7)	61 (24.6)	26 (10.5)
**Training**					
*Among those who received PI training:* “I feel adequately prepared to perform PI application after this training.”	99 (66.9)	33 (22.3)	12 (8.1)	4 (2.7)	0
**Resources and Application**					
“I have all the supplies I need to apply intranasal PI readily available.”	62 (25.0)	75 (30.2)	24 (9.7)	60 (24.2)	27 (10.9)
“I am able to consistently perform nasal decolonization according to steps outlined in the SOP.”	44 (17.8)	77 (31.2)	50 (20.2)	54 (21.9)	22 (8.9)
“I am able to routinely swab both nostrils for 30 seconds each.”	51 (20.6)	72 (29.0)	39 (15.7)	59 (23.8)	27 (10.9)
“I am easily able to swab PI in the nostrils even if there are other nasal devices in place (eg, nasal intubation, NG tube)”	34 (13.7)	60 (24.2)	51 (20.6)	73 (29.4)	30 (12.1)
“Even with other patient duties, I have enough time to complete the recommended doses of intranasal PI for my patients.”	47 (19.0)	78 (31.6)	56 (22.7)	43 (17.4)	23 (9.3)
**Patient Acceptance**					
“Patients are willing to have intranasal PI performed.”	4 (1.6)	51 (20.6)	46 (18.6)	80 (32.4)	66 (26.7)
“Patients generally tolerate intranasal PI well without complications.”	19 (7.7)	101 (40.9)	54 (21.9)	44 (17.8)	29 (11.7)

Note. PI, povidone-iodine; SOP, standard operating procedure.

## Results

Among 248 respondents from a pool of ∼1,000 nurses, most were new to nursing (54.4% had 0–4 years of experience) and worked in non-ICU units (61.5% vs. 38.5% in ICUs). Overall, 150 respondents (60.5%) reported receiving training on the intranasal PI SOP: hands-on training (48.6%), electronic module training (25.7%), or both types of training (20.9%). Nurses who reported training indicated moderate to strong confidence in their ability to perform intranasal PI decolonization (89.2%). Most respondents cited a good understanding of the rationale for use and appropriately identified patients who required decolonization (Table 1). Minimal time was spent performing intranasal PI, with 81.3% of respondents indicating the full process took less than 2 minutes.

Among all respondents, 49% were able to consistently perform decolonization as outlined in the SOP. Barriers included inadequate supplies (35.1%), lack of a readily available copy of the SOP (69%), difficult application in patients with nasal devices in place (41.5%), and time constraints from other patient duties. While 49.2% of nurses agreed or strongly agreed that patients understood why PI decolonization was performed, only 22.2% of nurses agreed or strongly agreed that patients were generally willing to undergo decolonization. Other challenges included difficulty tracking PI application within the electronic medical record (EMR), limited knowledge of PI effectiveness, patient refusal despite education, and overall personnel burnout. Compared with non-ICU nurses, ICU nurses were significantly less confident in their ability to identify patients who required decolonization (*P* = .002), communicate PI use with patients (*P* = .006), and perceived significantly lower patient comprehension of PI *(P =* .025) (Table 2).

**Table 2.** Frontline Nursing Survey Results on Barriers to Povidone-Iodine Nasal Decolonization Protocol Adherence Stratified by ICU versus non-ICU Unit-Based Respondents

Survey Question	ICU (N=95)	Non-ICU (N=152)	*P* Value
“How many years have you been in this [nursing] role?”	0-4 years (52 = 54.7%) >20 years (5 = 5.3%)	0-4 years (83 = 54.6%) >20 years (21 = 13.8%)	.988 **.0003**
	Respondents Who Indicated Moderate-to-Strong Agreement, No. (%)		
“I have a good understanding as to why we use intranasal PI on our patients.”	69 (72.6)	122 (80.3)	.159
“I can easily identify which of my patients need to undergo intranasal PI.”	55 (57.9)	116 (76.3)	**.002**
“I am comfortable talking with patients who have questions about the PI application.”	59 (62.1)	119 (78.3)	**.006**
“My patients understand why intranasal PI decolonization is being performed.”	38 (40.0)	83 (54.6)	**.025**
“I have all the supplies I need to apply intranasal PI readily available.”	47 (49.5)	89 (58.5)	.167
“I am able to consistently perform nasal decolonization according to steps outlined in the SOP.”	36 (37.9)	84 (55.3)	.374
“I am able to routinely swab both nostrils for 30 seconds each.”	41 (43.2)	81 (53.3)	.105
“I am easily able to swab PI in the nostrils even if there are other nasal devices in place (eg, nasal intubation, NG tube).”	33 (34.7)	60 (39.4%)	.458
“Even with other patient duties, I have enough time to complete the recommended doses of intranasal PI for my patients.”	40 (42.1)	85 (55.9%)	**.035**
“Patients are willing to have intranasal PI performed.”	16 (16.8)	38 (25.0)	.129
“Patients generally tolerate intranasal PI well without complications.”	39 (41.0)	80 (52.6)	.076

Note. PI, povidone-iodine; SOP, standard operating procedure. Bold indicates statistical significance.

## Discussion

Universal nasal decolonization for high-risk patients is a core strategy to reduce *S. aureus* HAIs.^8^ Implementation barriers to decolonization policies have not been well characterized, particularly from the nursing perspective.

During the development of the SOP, certain logistics were prioritized to limit barriers to use. In addition to lower cost and risk of resistance, PI was selected over mupirocin because it did not require a provider order as an intranasal antiseptic. Adherence rates to the SOP, however, were suboptimal after initial implementation, likely exacerbated by personnel and resources shortages during the COVID-19 pandemic. Frequent staffing turnover in ICU units during pandemic peaks likely contributed to greater barriers perceived by ICU nurses regarding decolonization practices. Low training rates may have been influenced by the need to focus education on care of COVID patients, particularly among newly hired nurses.

Insufficient understanding of the rationale for PI use, including potential risks of not performing decolonization, was the leading concern expressed in free-text comments. This gap in training was compounded by challenges with supply availability at the bedside and aspects of the PI product. Specifically, the large swab size was noted to impede intranasal application, with concerns for transient desaturation if supplemental oxygen devices were in place as well as dissatisfaction with possible brown discoloration of nares. Although most respondents indicated that they could identify which patients required PI and were comfortable communicating the reasoning for decolonization, they perceived that most patients declined despite education. Even if patients were agreeable, PI supply was limited and tracking doses on the EMR was challenging, as PI was not included in the medication administration record (MAR). Instead, nurses relied on electronic triggers from an EMR task tool separate from the MAR, which many cited as a difficult platform to navigate, leading to missed or extra doses.

These results highlight opportunities to improve nurse and patient education on decolonization practices and EMR tracking logistics. Similarly, a study by Hammond *et al.* ^9^ evaluated surgical nursing perspectives of implementation barriers with a nasal PI protocol to prevent surgical site infections. Their findings highlighted the significance of education and training in pre-implementation stages to optimize practice technique, promote patient understanding, and engage nursing leaders in protocol development.^9^ Educational resources should include hands-on training sessions and electronic modules to reinforce aims and benefits of use, and provide easily accessible references for the SOP. Collaboration with nursing partners in focus groups and designated unit champions will strengthen these initiatives and provide feedback to inform protocol development. Education is necessary but alone is insufficient to change practice. Integration of nasal decolonization into EMR order sets for ICU admissions, transfers, and after placement of CVC or midline catheters should also be prioritized. Ultimately, product selection should be revisited based on the barriers identified in our survey and new data showing superiority of mupirocin over iodophor (both with CHG bathing for 5 days) in reducing rates of ICU *S. aureus* and MRSA clinical isolates.^10^

This study had several limitations. It may have been subject to potential negative bias by nurses experiencing burnout during the COVID-19 pandemic. Survey response rates improved after sending a second e-mail link, though overall were low (25%) and may have been driven by burnout. Perceived barriers may differ by specific ICU and non-ICU units, type of training received, and experience in the nursing role, which this study was not powered to determine. Nevertheless, these results provide insight into nursing barriers to intranasal decolonization utilization. Optimization of nasal decolonization SOP adherence and prevention of *S. aureus*–associated HAI requires further understanding of nursing barriers regarding education, logistics, and aspects of the antiseptic product. Our findings underscore the need to develop and disseminate educational resources for nurses and patients to improve protocol fidelity and acceptance.
